# Endophytic *Streptomyces* population induced by L-glutamic acid enhances plant resilience to abiotic stresses in tomato

**DOI:** 10.3389/fmicb.2023.1180538

**Published:** 2023-06-09

**Authors:** Da-Ran Kim, Youn-Sig Kwak

**Affiliations:** ^1^Research Institute of Life Science, Gyeongsang National University, Jinju, Republic of Korea; ^2^Division of Applied Life Science (BK21 Plus), Research Institute of Life Science, Gyeongsang National University, Jinju, Republic of Korea

**Keywords:** drought stress, endosphere, prebiotic, probiotic, salinity stress

## Abstract

Endophyte bacteria, which colonize plants including roots, stem, flower, and fruit, it can derive their nutrients from the host, are recognized for their mutualistic relationship with the host plant. They play a critical role in promoting host growth and modulating abiotic stress. Carbon and nitrogen have a significant impact on bacterial population and secondary metabolite production, which are highly specific in various categories such as bacterial growth regulation, anti-compounds production. Application of L-glutamic acid can significantly enhance *Streptomyces globisporus* population buildup in plants. However, the effectiveness of this population buildup against abiotic stresses such as salinity and drought has not been investigated. Therefore, in this study, we tested the bacteria and their prebiotic activity against salinity and drought stress in tomato plants. Three different amino acids were treated on the tomato plants, and it was observed that L-asparagine and L-proline had a negative effect on plant growth and phenotype, while L-glutamic acid promoted plant growth and increased bacteria population density. The bacteria were found to colonize the rhizosphere and root endosphere, with colonization being promoted by L-glutamic acid. Additionally, *Streptomyces* was found to have plant growth promotion effects and provided protection against abiotic stresses. Interestingly, L-glutamic acid reduced the damage caused by salinity stress, but not drought stress. These findings suggest that L-glutamic acid plays a role in providing tolerance to salinity stress with the core microbiota, thus the current study demonstrated their prebiotic activity in the agriculture system.

## Introduction

Climate change and global warming are causing extreme stresses on plants, such as droughts and salt stress, which have severe effects on agricultural production (Pawlak and Kołodziejczak, 2020; Fasusi et al., 2021). Environmental abiotic stresses have also reduced crop productivity, and currently, no chemical or cultivation method can completely prevent this. However, plant growth-promoting rhizobacteria (PGPR) can partially reduce damage caused by abiotic stresses (Berkelmann et al., 2020). PGPRs have various plant growth enhancement abilities, such as IAA (Indole-3-acetic acid) production, nitrogen fixation, and siderophore production (Herrera-Quiterio et al., 2020; Andleeb et al., 2022). Certain genera of bacteria, such as *Bacillus*, *Pseudomonas*, and *Streptomyces* are known as PGPR functional bacteria with direct or indirect mechanisms to enhance plant growth (Bokhari et al., 2019; Fidan and Zhan, 2019; Oviedo-Pereira et al., 2022).

Recent interest in plant endophytic bacteria has focused on their impact on host physiology. These bacteria thrive in the endosphere of plants, especially the nutrient-rich endodermis xylem tissue. The relationship between endophytic bacteria and their hosts has evolved into a symbiotic one, with the bacteria relying on the plant for nutrients (Pacifico et al., 2019; Lacava et al., 2022). Understanding this interaction has the potential to inform our knowledge of plant-microbe interactions and their implications for ecosystem function. Among them, *Streptomyces* is a beneficial endo-microorganism, producing bioactive compounds that protect crops such as wheat, rice, tomato, and strawberry, it has produces a variety of novel bioactive compounds, such as kakadumycin, coronamycin, grisin, and conprimycin (Ezra et al., 2004; Cha et al., 2016; Goudjal et al., 2016; Phuakjaiphaeo et al., 2016; Colombo et al., 2019; Kim et al., 2019a; Xu et al., 2019; Mattei et al., 2022). These secondary metabolites have antibacterial and antifungal properties, enhancing growth and protecting against biotic and abiotic stress, with have antifungal to *Fusarium*, *Alternaria*, and *Pyricularia* and antibacterial to *Pseudomonas syringae* and in abiotic stress related drought and salinity (Wang et al., 2010; Cha et al., 2016; Passari et al., 2017; Jeon et al., 2019; Kim et al., 2019a; Nozari et al., 2022). However, some of the metabolites remain unstudied, indicating a need for further research on the potential of *Streptomyces* to improve plant health and productivity.

Growth of *Streptomyces* can be enhanced by specific nutrient sources under natural conditions, as observed in the host plants (Costa et al., 2002; Reese et al., 2018; Wang et al., 2019; Estrela et al., 2021). In human microbiology, such nutrients are referred to as prebiotics, which regulate and modulate metabolic pathways (Ramnani et al., 2015; Samanta et al., 2015; Yang et al., 2021). Similarly, in plants, certain nutrients can support and modulate microbial growth and population density, not only enhancing probiotic effect to cell growth but also contributing significantly to the microbe’s functional interaction with the host (Ducray et al., 2019; Du et al., 2021). Bacteria require nutrients for the production of enzymes, capsules, and colonization, and nitrogen compounds from plant origins are favorable nutrients for the colonizing bacteria. These compounds are utilized in the production of bioactive compounds and proteins with antibiotic activity (Voelker and Altaba, 2001; Costa et al., 2002; Bibb, 2005; Pandey et al., 2005; Rico and Preston, 2008). Prebiotics can also modulate bacterial growth in floral nectar and rhizosphere, serving as signal molecules for plant growth-promoting activity in bacteria (Borghi and Fernie, 2017; Aliashkevich et al., 2018). Kim et al. (2021) reported that L-glutamic acid can rebuild the population of a core bacterium strain in both above-ground and below-ground plant tissues, reshaping microbial community structures, maintaining high microbial diversity, and preventing plant pathogen attack. Moreover, the rebuilt core strain can move from root to stem and from flower to root tissue through the endosphere.

Tomatoes have been found to harbor a beneficial endophytic *Streptomyces* strain, which interestingly, is genetically identical to the strain found in other plant species (Cho and Kwak, 2019). Tomatoes are subjected to a range of abiotic stresses such as drought, salinity, osmotic and ionic stresses, exacerbated by climate change. Several studies have demonstrated that the use of engineered bacterial communities can increase the accumulation of proline, a known osmoprotectant, in tomato plants under drought and salt stress conditions (Palaniyandi et al., 2014). Additionally, the presence of endophytic *Streptomyces* populations can improve the plant’s capacity to absorb nutrients and maintain a healthy system, which further enhances its resilience to environmental stresses (Niu et al., 2022).

This raises the question of how this microbe is able to provide benefits to multiple host plants through its endophytic colonization system. Furthermore, it is unknown whether a probiotic strain that can regulate the microbial community structure and increase the density of the core strain in strawberries can also exhibit the same effect in other plant systems. In this study, we aim to investigate the colonization and modulation of the functional core *Streptomyces* strain by L-glutamic acid in tomato plants, as well as the bacterial efficacy in protecting against abiotic stresses.

## Materials and methods

### Plant growth condition

The surface of tomato (cv. Heinz) seeds was sterilized by immersing them in 70% ethanol for 5 min followed by 1% NaOCl for 3 min. The seeds were then washed twice with ddH_2_O in a 50 ml tube and centrifuged at 4,000 rpm for 5 min to remove any dust. The supernatant was discarded using a 90-mm filter paper on a glass funnel. The sterilized seeds were air-dried at room temperature for 30 min in an air-clean biosafety cabinet. The dried seeds were then placed on water-soaked cotton for germination at 25°C for 3 days and subsequently planted in plastic pots (9 cm × 7 cm) filled with nursery soil. The pots were incubated in a plant growth chamber from week 2 to week 6, with the following conditions: 25°C for 20 h under light (13,000 lux) and 22°C for 4 h in the dark with 80% relative humidity.

### *Streptomyces* strains and seed coating

Tomato (cv. Heinz) seeds were sterilized by immersing them in 70% ethanol and 1% NaOCl solution, followed by drying in a biosafety cabinet for 30 min. *Streptomyces* strains were isolated from various host plants, including strawberry (SP6C4::hyg), kiwifruit (KPB2::hyg and W1SF4::hyg), and turfgrass (S8::hyg) (Table 1). The bacteria were grown in 5 ml of PDK broth media (10 g potato dextrose and 10 g peptone per L) at 30°C for 5 days in a shaking incubator (150 rpm). The cultured bacteria were then transferred to 500 ml of PDK broth media and main culture at 30°C for 10 days in the shaking incubator. The bacteria were washed out of media nutrients by centrifugation for 15 min and resuspended in 50 ml of ddH_2_O (10^6^ cfu/ml) with 1% carboxymethyl cellulose (CMC). Tomato seeds were dipped in the bacterial strain cultured stocks, and control untreated seeds were soaked in ddH_2_O (*n* = 5, each plant was a different replicate). The tomato plants received watering twice a week with 10 ml of 1X Hoagland’s solution, which contained macronutrients [101.11 g of KNO_3_ (1 M), 115.03 g of NH_4_H_2_PO_4_ (1 M), 236.15 g of Ca (NO_3_)_2_⋅4H_2_O (1 M), 246.48 g of MgSO_4_⋅7H_2_O (1 M), 0.5% (w/v) Fe-EDDHA per L], micronutrients [2.86 g of H_3_BO_3_, 1.81 g MnCl_2_⋅4H_2_O, 0.22 g of ZnSO_4_⋅7H_2_O, 0.051 g of CuSO_4_⋅5H_2_O, 0.09 g of Na_2_MoO_4_⋅2H_2_O per L added 33 ml of 15% NaCl solution, 20% (w/v) PEG6000 and 200 mM NaCl solution] (Renau-Morata et al., 2014).

**TABLE 1:** List of *Streptomyces* strains in this study.

Bacterial identification	Host	Origin	References
*Streptomyces parvulus* KPB2	Kiwifruit	Phyllosphere (pollen)	Kim et al., 2019b
*Streptomyces racemochromogenes* W1SF4	Kiwifruit	Rhizosphere	Kim et al., 2019b
*Streptomyces globisporus* SP6C4	Strawberry	Phyllosphere (pollen)	Kim et al., 2019a
*Streptomyces bacillaris* S8	Turfgrass	Rhizosphere	Jeon et al., 2019

### Colonization of the *Streptomyces* strains in rhizosphere and endosphere of tomato

Seed-coated tomato plants containing KPB2::hyg, W1SF4::hyg, SP6C4::hyg, or S8::hyg were used to determine the bacterial population densities in the rhizosphere and endosphere, as well as the contents of chlorophyll and fresh weight of tomato. Two weeks after the treatments, rhizosphere bacterial cells were collected by placing 1 g of root with rhizosphere soil in 2.5 mM MES [Monohydrate 2-(N-Morpholino)ethanesulfinic acid] buffer (pH 5.7 adjusted with 1M KOH) in a 15 ml tube, which was then vortexed for 15 sec and incubated at room temperature for 15 min. The mixture was centrifuged at 500 rpm for 5 min and the supernatant was transferred to a new 1.5 ml tube, which was then centrifuged at 8,000 rpm for 15 min. The supernatant was discarded, and the pellet was resuspended in 1 ml MES buffer (2.5 mM). The resuspended samples were serially diluted with ddH_2_O up to 10^–8^ and 100 μl was spread on PDK media [PDK: Potato Dextrose Broth (BD Difco, Franklin Lakes, NJ, USA) 10 g, Peptone 10 g, Agar 20 g per L] with hygromycin B (final concentration: 80 μg/ml). The plates were incubated at 28°C for 5 days to determine the colony-forming units (cfu).

To detect colonization of the *Streptomyces* strains in the endosphere, bacterial cultures were grown in flasks at 30°C for 10 days in a shaking incubator. The bacterial stock was then mixed with CMC (carboxymethyl cellulose) to achieve a final concentration of 0.1% v/v, and the resulting solution was used to drench tomato plants during the growth stage. Each plant received 10 ml of the bacterial mixture. After 2 weeks, tomato shoot length and weight were recorded, and rhizosphere, root, and stem samples were collected. To isolate endophytic bacteria, the roots, and stems were washed twice in ddH_2_O and added to a 50-ml tube, which was sonicated at 35 MHz for 5 min in 1X PBS (Phosphate-Buffered Saline, prepared by adding 80 g of NaCl, 2 g of KCl, 4.4 g of Na_2_HPO_4_⋅2H_2_O, 2.4 g of KH_2_PO_4_ per L and adjusting the pH to 7.4). This step was performed to remove surface soil and unwanted microbes. Surface sterilization was then carried out by treating the tissue samples with 70% ethanol for 2 min, followed by 1% NaOCl for 3 min, and washing twice with ddH_2_O. The sterilized tissue samples (0.5 g) were ground using a mortar and pestle and filtered through a single layer of cheesecloth into a 1.5-ml tube containing 1 ml of 1X PBS. The resulting mixture was diluted up to 10^–4^ with ddH_2_O, and 100 μL of the diluted mixture was spread onto PDK plates supplemented with hygromycin B (final concentration: 80 μg/ml) to isolate the endophytic microbes. For the rhizosphere samples, 9 ml of MES buffer was added, and serial dilutions were made up to 10^–8^ using ddH_2_O. The diluted samples were then spread onto PDK media, and the plates were incubated at 28°C for 5 days. Colony-forming units were counted to quantify the endophytic microbial population.

### Amino acids treatment of tomato

A stock solution of L-asparagine, L-proline, and L-glutamic acid (known to have *Streptomyces* growth-promoting effects) (Kim et al., 2021) was prepared at a concentration of 5 μg/ml. L-asparagine and L-proline were solubilized in ddH_2_O after autoclaving, while L-glutamic acid was added to ddH_2_O, and its pH was adjusted using 1M KOH. Tomato plants were grown for 20 days in plastic pots. Ten ml of the amino acid solutions were administered to each tomato plant once a week for 3 weeks (*n* = 10), with plants receiving different treatments being separated in separate plastic trays. A combination of the SP6C4 strain of *Streptomyces globisporus* and L-glutamic acid was applied to different plastic trays and drenched onto plants 2 days before the amino acid treatment.

### Glutamic acid and SP6C4 application with drought stress

Tomato plants (cv. Heinze) were grown under controlled conditions of 24°C for 16 h and 26°C for 8 h, with 80% relative humidity for 2 weeks. The plants were grown separately in plastic trays, and treated with either L-glutamic acid or SP6C4::hyg. The L-glutamic acid pH was adjusted with 1M KOH, and the bacterial stock of SP6C4 was prepared with 1% CMC (10^6^ cfu/ml), which was then applied to the tomato plants (10 ml) at 7-day intervals. The treatments included: untreated (*n* = 20), drought stress (*n* = 20), L-glutamic acid with drought stress (*n* = 20), and SP6C4::hyg with drought stress (*n* = 20). Drought conditions were applied to 20 plants for each treatment, with 10 plants used for recovery treatment. The amino acids were supplied twice, after 3 days and at 2 weeks. The untreated plants were supplied with 1X Hoagland solution every 3 days prior to the drought stress. To induce drought stress, the water supply was stopped for 7 days, and then 1X Hoagland solution was supplied during the recovery stage for 2 days to each plant, with each plant receiving 10 ml of the solution. On the 28th day (the last day under drought stress) and on the 30th day (after the recovery treatment), the plants were measured for shoot length, weight, and stomatal aperture.

### Glutamic acid and SP6C4 treatments for salt stress

A salinity stress experiment was performed using 2-week-old tomato plants (cv. Heinz) with 4–6 leaves, grown under conditions of 25°C for 16 h (light) and 22°C for 8 h (dark), and 80% relative humidity. Twenty plants were used for each treatment, with ten plants per replicate after stress and the other ten for the recovery stage. The treatments comprised seven conditions: untreated control, 75 mM NaCl, 100 mM NaCl, L-glutamic acid, L-glutamic acid with 75 mM NaCl and 100 mM NaCl, SP6C4 (10^6^ cfu/ml), and SP6C4 with 75 mM NaCl and 100 mM NaCl. Salinity stress was induced by drenching the plants with 10 ml of 75 mM or 100 mM NaCl solutions in week 4, and the plants were collected in week 5 after the stress treatments. The recovery samples were resupplied with 1X Hoagland solution with 10 ml for 1 week. The shoot length and plant weight were measured after the salt stress treatments and the recovery stage.

### Tomato microbiota analysis

Tomato tissues were collected for the analysis of the microbiota community structure. The root samples were washed with 70% EtOH for 30 sec, 1% NaOCl for 30 sec, and rinsed with ddH_2_O two times, followed by sonication with 50 ml of 1X PBS buffer for 15 min. DNA extraction was performed using the soil FastDNA SPIN Kit (MP Biomedicals, Santa Ana, CA, USA) with 0.5 g of root samples, 122 μL of MT buffer, 978 μL of sodium phosphate buffer, and homogenized with FastPrep-24TM Classic bead homogenizer (MP Biomedicals, Santa Ana, CA USA) following the manufacturer’s instructions. The extracted DNA was washed with 500 μL of SEWS-M buffer with EtOH and centrifuged at 14,000 *g* for 1 min. The catch tube was discarded, and a membrane filter was centrifuged at 14,000 *g* for 2 min to remove the wash buffer. The filter was then added with 50 μL of DES buffer, and the DNA quantity and quality were calculated with a spectrometer (Nanodrop 2000C, Thermo Scientific, Waltham, USA). The extracted DNA (10∼20 ng/μL) was used for blocking PCR, which is a necessary step to block plant plastid DNA amplicon chimera (Kim et al., 2021). The endophyte microbial pyrosequencing PCR step was performed with 20 μL of KAPA HiFi HotStart ReadyMix (Roche, Basel, Switzerland), PNA (5′-GGCTCAACCCTGGACAG-3′), mPNA (5′-GGCAAGTGTTCTTCGGA-3′), 515F primer (5′ TCGTCGGCAGCGTCAGATGTGTATAAGAGACAGG TGCCAGCMGCCGCGGAA-3′), and 805R primer (5′-GTCTCGTGGGCTCGGAGATGTGTATAAGAGACAGGACTAC HVGGGTATCTAATCC-3′). The PCR conditions were as follows: initial denaturation at 98°C for 5 min, followed by 22 cycling steps consisting of denaturation at 98°C for 1 min, PNA at 78°C for 10 sec, annealing at 55°C for 30 sec, extension at 72°C for 1 min, and final extension at 72°C for 5 min (Cho et al., 2022). To avoid amplification of the mitochondria and chloroplast, the PCR fragment was purified with an SV kit (GeneAll, Seoul, Republic of Korea), and the 16S rRNA partially V4 region pyrosequencing was performed with Illumine Miseq (2 bp × 300 bp) by Macrogen (Seoul, Republic of Korea).

The detached barcode sequences obtained from pyrosequencing were subjected to a quality check in FASTQ format. Two sequences with a forward length of 240-bp and reverse length of 200-bp were merged, and the resulting amplicon sequence variants (ASVs) were clustered using DADA2 (version 1.20) in R (version 4.2.1) as described by Callahan et al. (2016). Taxonomic assignments were performed using the IDTAXA classifier in the DECIPHER package (Version 2.20) with SILVA 138 SSU for each ASV, as described by Murali et al. (2018). Bacterial community analysis was performed using alpha diversity, bar plots of bacterial composition, and principal coordinates analysis (PCoA) using the phyloseq package (version 1.36). Results were visualized using ggplot2 (version 3.3.6) in R.

### Statistical analyses

The statistical analyses of all graphs were conducted using ANOVA, *t*-test, and Kruskal–Wallis tests. If the data did not meet the assumptions of normality and equal variance, they were analyzed using ANOVA and Kruskal–Wallis tests. *Post-hoc* tests were performed using Tukey’s HSD and pairwise Wilcox tests. The graphs were visualized using ggplot2 from the R software package.

## Results

### *Streptomyces* spp. colonization and growth promotion

We investigated the effects of four *Streptomyces* strains on tomato plants. The strains were applied through drenching, and their impact on chlorophyll content and fresh weight were evaluated. Results showed that chlorophyll content did not differ significantly between the untreated control, KPB2, and W1SF4 treatments. However, S8 and SP6C4 treatments led to a significant increase in chlorophyll content, more than double (400 mg^–1^ FW) that of the untreated control (200 mg^–1^ FW) (Supplementary Figure 1A). Similarly, fresh weight was found to be higher in S8 and SP6C4 treatments compared to the untreated control, KPB2, and W1SF4 treatments (Supplementary Figure 1B). Colonization of the tomato rhizosphere by the *Streptomyces* strains was observed at levels between 10^3^ and 10^4^ cfu/g of soil (Supplementary Figure 1C). Among the tested strains, only SP6C4 showed relatively high activity in PGPR and colonization, and thus further investigation was focused on this strain.

**FIGURE 1:**
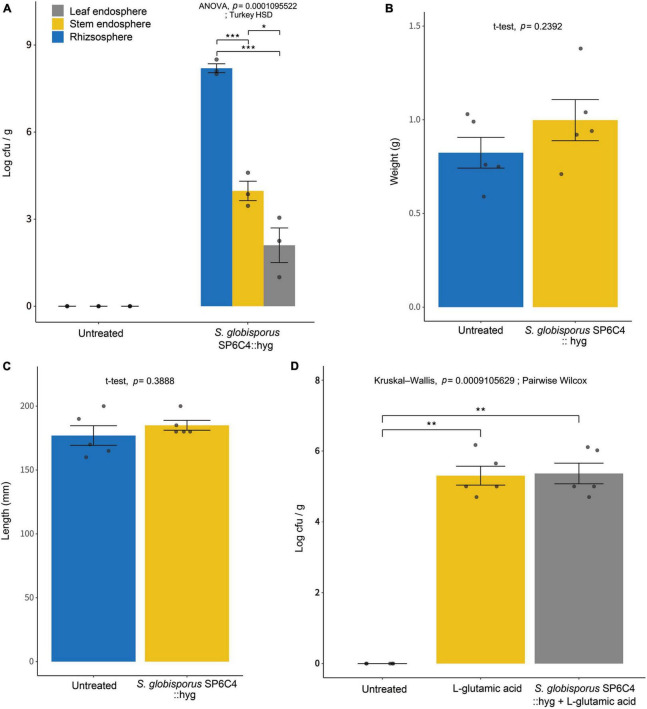
Effect of L-glutamic acid enriched *Streptomyces globisporus* SP6C4 on population density in the rhizosphere and endosphere of tomato plants (cv. Heinz). The plants were sterilized with 70% ethanol and 2% NaOCl, and then planted in pots. After 2 weeks, *S. globisporus* SP6C4::hyg 10^6^ cfu/ml with 1% carboxymethyl cellulose (CMC) was applied to the plants (*n* = 5). **(A)** The population density of *S. globisporus* SP6C4 was determined in the rhizosphere, stem endosphere, and leaf endosphere. Rhizosphere soil was serially diluted and endosphere tissue was washed with sonicated 1X PBS buffer for 15 min. **(B)** The tomato shoot weight was determined 2 weeks after treatment, **(C)** shoot length was measured. All plants were grown in a plant chamber at 28°C for 20 h of light and 22°C for 4 h of dark, with 80% relative humidity. Each bar on the graph represents the standard deviation with *p* = 0.05. **(D)** *Streptomyces* population density in the root endosphere was measured following treatment with L-glutamic acid (5 μg/ml) once per week from week 3 to week 6, with each tomato receiving 10 ml of the solution (*n* = 10). Statistically significant differences among treatments (*p < 0.05, **p < 0.01, ***p < 0.001).

After the application of the SP6C4::hyg strain on tomato plants for 7 days, bacterial population densities were determined on antibiotic-amended media. Results revealed population densities of 3.1 × 10^7^ cfu/ml, 5.6 × 10^5^ cfu/ml, and 5.8 × 10^3^ cfu/ml in the rhizosphere, stem endosphere, and leaf endosphere, respectively (Figure 1A). Furthermore, the application of *S. globisporus* SP6C4 significantly improved the growth of tomato plants. An increase in shoot weight and length was observed upon SP6C4 application (Figures 1B, C). Notably, among the three amino acids tested, L-glutamic acid treatment exhibited the most pronounced growth-promoting effect (Supplementary Figure 2). Combination of SP6C4 and L-glutamic acid treatment further enhanced plant growth. Moreover, L-glutamic acid treatment facilitated the growth of SP6C4 population in the tomato root endosphere (Figure 1D). Conversely, L-asparagine and L-proline treatment exerted negative effects on the host growth, causing damage to both above and below-ground tissues.

### SP6C4 enhanced drought stress tolerance

**FIGURE 2:**
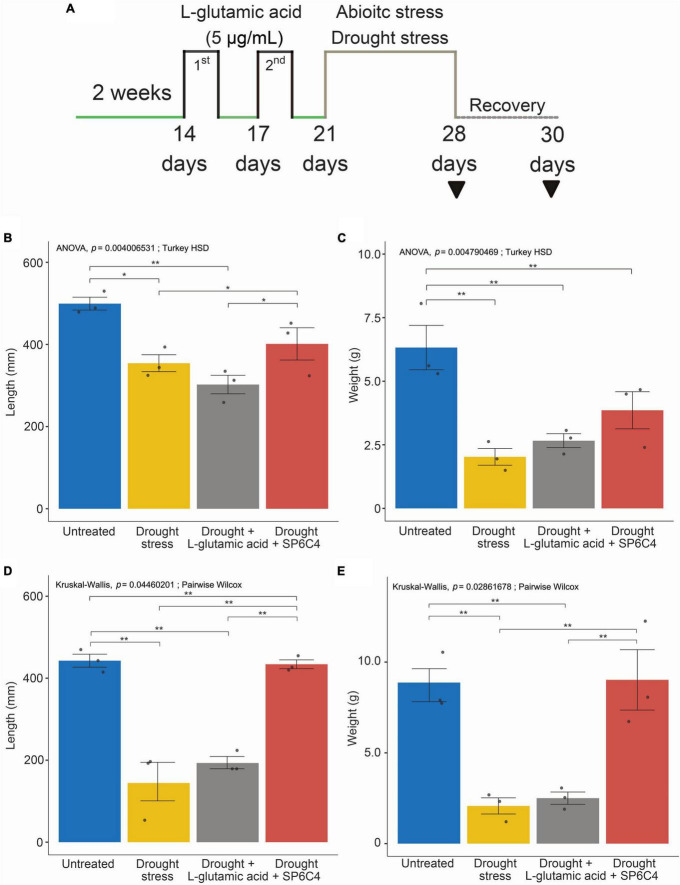
Phenotypic changes of tomato under normal and drought stress conditions. **(A)** Tomato (cv. Heinz 1350) shoot length and weight after drought stress and recovery during growth. The tomato was in the vegetative stage for 2 weeks, and L-glutamic acid (5 μg/ml) was applied with 10 ml per plant (*n* = 20). L-glutamic acid was added twice, followed by drought stress for 7 days, while untreated plants were watered with 1X Hoagland solution. For recovery, 1X Hoagland solution was supplied. **(B,C)** Plants under drought stress. **(D,E)** Plants under recovery condition. The bar on each graph indicates the standard deviation. A significant difference was observed at *p* = 0.05. Statistically significant differences among treatments (*p < 0.05, **p < 0.01).

**FIGURE 3:**
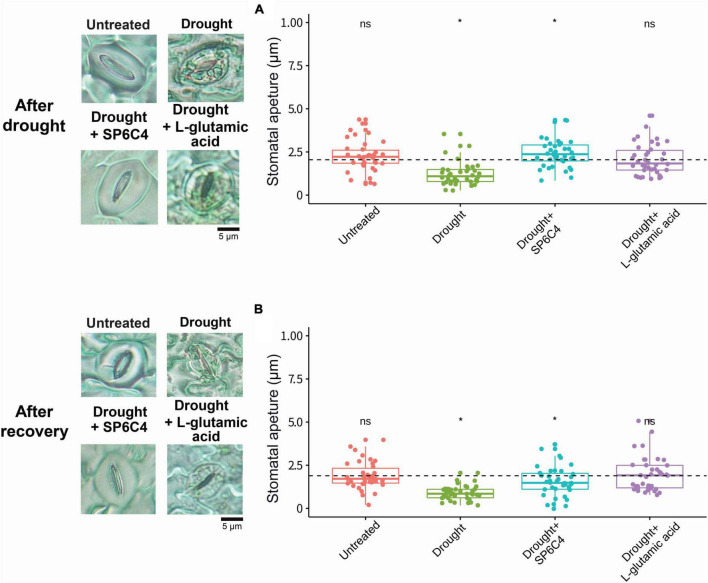
Tomato stomatal aperture during drought stress and recovery stage. Microscope images were captured to measure the stomatal aperture. **(A)** Representative image shows the stomatal aperture in four different treatments. **(B)** Stomatal aperture size was measured with a 100X magnification microscope after recovery with 1X Hoagland solution. Statistical significance was determined using ANOVA with a *p*-value of 0.05. Statistically significant differences among treatments (*p < 0.05).

L-glutamic acid was applied to 2-week-old tomato plants, followed by exposure to drought stress for 7 days (Figure 2A). Under drought stress conditions, the abiotic stress significantly suppressed tomato growth, including shoot length and fresh weight. Treatment with *S. globisporus* SP6C4 provided effective protection against drought stress (Figures 2B, C). However, L-glutamic acid treatment did not protect the plant against stress treatment. At the recovery stage, SP6C4-treated plants fully recovered from drought stress, including shoot length and fresh weight (Figures 2D, E). During exposure to drought stress, stomatal aperture closure and physical destruction occurred, but SP6C4-treated tomato plants maintained normal stomatal aperture and structure (Figure 3A). After recovery from drought stress, the stomatal aperture size was 2.0∼2.5 μm under normal conditions and SP6C4 treatment (Figure 3B). In contrast, damaged stomata had an opening size of 0∼2.0 μm and decolorized chlorophyll in the stomata cells. Interestingly, L-glutamic acid treatment also showed relatively less damage to stomatal shape both under drought conditions and after recovery. These findings suggest that SP6C4 may have a mechanism of stomatal aperture regulation-independent protection against drought stress.

### L-glutamic acid and SP6C4 protected tomato from salinity stress

**FIGURE 4:**
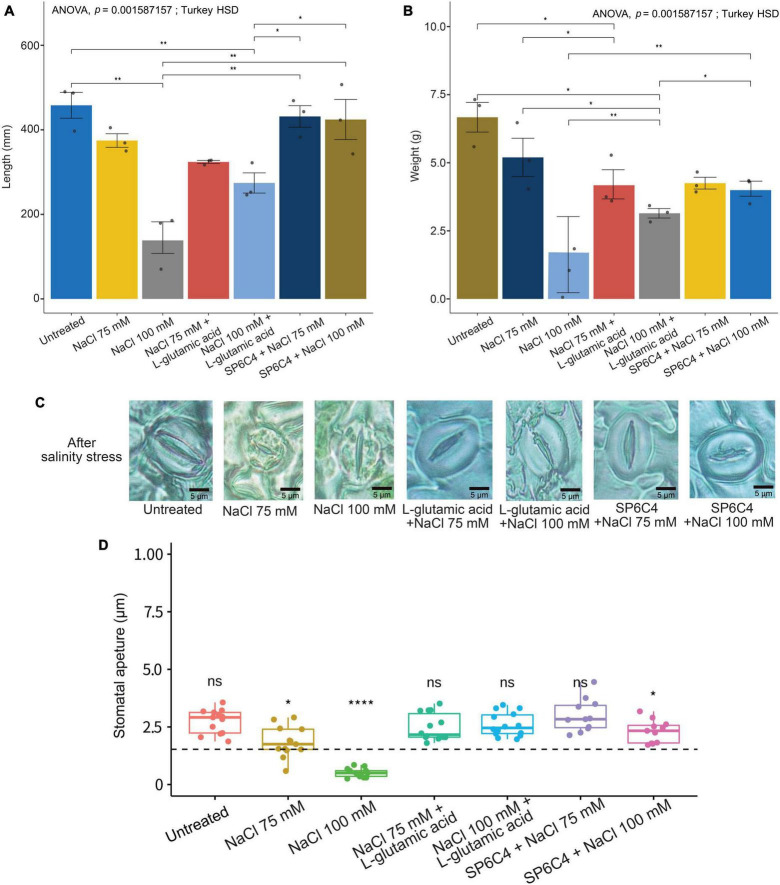
Salinity stress tolerance of tomato (cv. Heinz 1350) by SP6C4 and L-glutamic acid. **(A,B)** Show the above-ground phenotype changes of tomato plants under salinity stress for 14 days. The plants were treated with 75 mM and 100 mM NaCl, and L-glutamic acid was adjusted to pH 7.0 with KOH. **(C,D)** Demonstrate the stomatal aperture detected in the microscope (100X) with 75 mM NaCl and 100 mM NaCl only, while the other treatments were exposed to L-glutamic acid (5 μg/ml) or SP6C4 (10^6^ cfu/ml) in week 5. The figures were analyzed statistically with ANOVA and *post-hoc* test in Tukey HSD, with a *p*-value cut-off of 0.05. The data were collected from ten replicates. Statistically significant differences among treatments (*p < 0.05, **p < 0.01, ****p < 0.001).

The results demonstrated that exposure of tomato plants to 75 mM or 100 mM NaCl resulted in a significant reduction in shoot length and fresh weight (Figures 4A, B). Moreover, plants exposed to 100 mM NaCl exhibited visible symptoms of stress, including stunting, leaf twisting, and root browning. Treatment with the SP6C4 strain was found to reduce damage at the 100 mM NaCl concentration but did not show a significant effect at the 75 mM NaCl concentration, possibly due to the lower levels of damage observed at this concentration. Interestingly, L-glutamic acid treatment also resulted in a reduction in salinity damage at the 100 mM NaCl concentration, but not at the 75 mM NaCl concentration (Figures 4A, B). Analysis of stomata shape and aperture size revealed that untreated control, L-glutamic acid-treated, and SP6C4-treated plants maintained normal stomatal morphology, regardless of NaCl concentration. However, tomato plants exposed to 75 mM or 100 mM NaCl showed a significant reduction in stomata aperture size and shape (Figures 4C, D). These findings suggest that treatment with SP6C4 bacteria and L-glutamic acid may provide a potential strategy for mitigating salinity stress in tomato plants.

### Tomato endophytic bacterial community structure shifting by L-glutamic acid treatment

**FIGURE 5:**
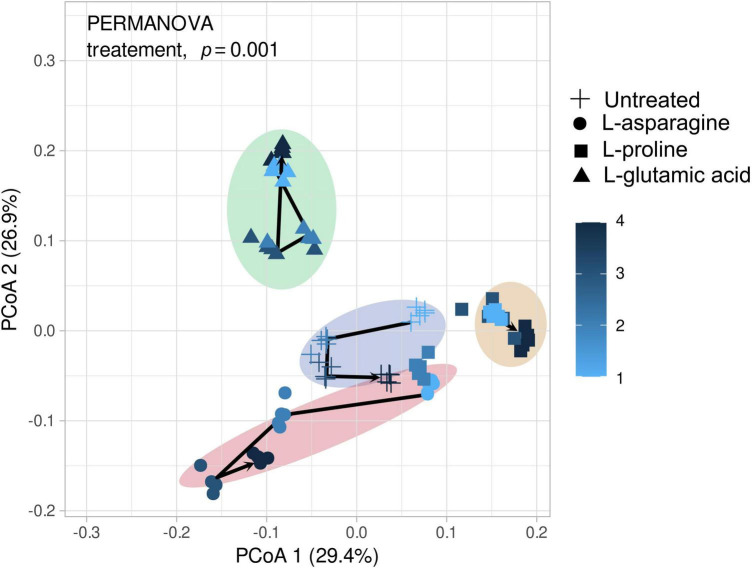
Depicts the microbial community changes following three different amino acid treatments. The microbial diversity was analyzed using the V4 region (250 bp) with Illumina Miseq and principal coordinate analysis (PCoA) to present similarities or dissimilarities based on the Bray–Curtis distance (*n* = 5). The different shapes in the figure represent the amino acid groups, while the color changes from bright to dark as the sampling time progresses. The visualized data were statistically analyzed by PERMANOVA.

**FIGURE 6:**
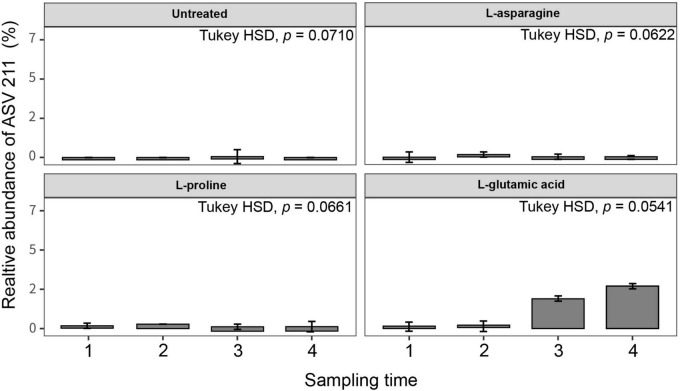
Relative abundance of *Streptomyces* ASVs in different amino acid treatments. Tomato plants were grown in soil treated with three types of amino acids (L-asparagine, L-proline, and L-glutamic acid) for 3 weeks (*n* = 10). The amino acid stock concentration was 5 μg/ml, with 10 ml drenched per pot per week. The root endosphere microbial community was analyzed using the 16S rRNA V4 region, and unique ASVs were identified using the base package (4.3 version) unique in R (4.2.1). Statistical analysis was conducted using Kruskal–Wallis and Tukey’s HSD tests in the treatment group (untreated, L-asparagine, L-proline, and L-glutamic acid).

Three different amino acids were used to treat tomato plants and their impact on the root endophytic bacterial community structure was investigated. The alpha diversity analysis of Shannon, Simpson, and Observed metrics showed that L-glutamic acid had the most significant effect on the root endophytic microbiota community structure, with each treatment displaying a unique pattern (Supplementary Figure 3). Interestingly, untreated tomato plants maintained a stable microbial diversity over the sampling periods. The beta diversity analysis revealed that Gammaproteobacteria was the most consistent group across all treatments. L-glutamic acid treatment resulted in an increase in specific bacterial taxa, including Streptomycetaceae and Burkholderiaceae (Supplementary Figure 4). Furthermore, the L-glutamic acid-treated tomato plants displayed distinct microbiota community patterns when compared to the untreated and other amino acids treated groups (Figure 5). Remarkably, only the L-glutamic acid-treated tomato plants showed an enrichment of the unique taxon *Streptomyces* ASV 211, which increased from 0 to 3.8% (Figure 6). These findings suggest that L-glutamic acid plays a vital role in promoting the growth of certain microbial members in the endosphere, which may be associated with plant growth promotion and protection against drought and salinity stresses.

## Discussion

Plants are confronted with a multitude of biotic and abiotic stressors that severely reduce their productivity (Mishra et al., 2015; Diagne et al., 2020; Arora and Fatima, 2022). These stressors, coupled with the effects of climate change, threaten the agricultural system by exposing crops to harmful factors such as drought and salinity. The symptoms of such stresses include leaf rolling and twisting, browning, and decreased yield (Li et al., 2022; Makhtoum et al., 2022). One promising approach to mitigate the impact of environmental stresses on plants involves leveraging the protective properties of certain plant-associated beneficial microbes (Worsley et al., 2020; Bhagat et al., 2021; Mohamad et al., 2022). *Bacillus* and *Burkholderia*, two genera of PGPR strains, are known to colonize the rhizosphere and endosphere of plants, protecting the host from abiotic stresses (Liu et al., 2017; Bokhari et al., 2019; Fidan and Zhan, 2019; Oviedo-Pereira et al., 2022). Additionally, endophytic *Streptomyces* spp. have been identified as probiotic strains in numerous crops (Pandey et al., 2005; Patel et al., 2018; Kim et al., 2019a,b; Donald et al., 2022). Kim et al. (2019a) reported that *S. globisporus* SP6C4, a core strain, aggressively colonized and modulated the whole microbiota structure in the strawberry root, stem endodermis, and flower. This strain was also capable of translocating from root to flower and vice versa through the plant vascular bundle system.

The SP6C4 stain has been shown to successfully colonize two different hosts, plants and insects, and exhibits anti-pathogen activity against a range of plant pathogens such as *Fusarium oxysporum* f. sp. *fragariae*, *Botrytis cinerea*, and *Cladosporium cladosporioides* (Kim and Kwak, 2021). Additionally, it also exhibits antibacterial activity against entomopathogens, *Paenibacillus larvae*, and *Serratia marcescens* (Kim et al., 2019a,^2021^). Interestingly, triple kingdom mutualism has been observed with this strain, whereby the populations in the ecosystem can be rebuilt by introducing L-glutamic acid. This presented the potential to engineer the microbiota community in both the anthosphere and rhizosphere of strawberry (Kim et al., 2021). Another strain, *S. globisporus* TFH56, isolated from tomato, has also shown a high degree of similarity with the SP6C4 strain at the whole genome scale (Cho and Kwak, 2019). However, the potential effects of this strain and glutamic acid against abiotic stresses, such as drought and salinity, remain to be explored.

The findings of this study demonstrated that SP6C4 enhances tolerance to both drought and salinity stresses in tomatoes when colonizing the rhizosphere and root endosphere. In contrast, L-glutamic acid was found to protect tomatoes specifically from salinity stress, and nitrogen sources were found to trigger changes in the microbiota. The microbiota structure included unique taxa that have been used as probiotics to reduce damage caused by salinity stress. Additionally, L-glutamic acid was found to decrease abiotic stress damage by increasing the population of *Streptomyces* and changing the microbial community structure, which are mechanisms that differ from those observed with L-asparagine and L-cysteine treatments. These results suggested that L-glutamic acid may be an essential nutrient for certain *Streptomyces* growth and population building in the microbiota. Nitrogen nutrients are also critical for living bacteria in a host and are essential to the microbial engineering system (Jonsbu et al., 2000; Costa et al., 2002; Reese et al., 2018; Wang et al., 2019; Estrela et al., 2021; Lau et al., 2022). Some nitrogen plays a role in signal molecules to improve pathogenicity as biofilm and communication molecules, which is a common strategy for biological function (Ramnani et al., 2015; Samanta et al., 2015; Yang et al., 2021). However, these functions and biological roles in the ecosystem are largely unexplored. Our study highlights the distinct biological role of L-glutamic acid in changing microbial communities and controlling salinity stress and may inform future efforts to engineer bacterial communities or core microbe properties to promote host tolerance to abiotic stresses.

## Data availability statement

The data presented in this study are deposited in the NCBI repository, accession number: PRJEB61093.

## Author contributions

D-RK performed experiments and data analyses. D-RK and Y-SK designed the research and wrote the manuscript. Both authors contributed to the article and approved the submitted version.
