# Supplementary material for: Endophytic Streptomyces population induced by L-glutamic acid enhances plant resilience to abiotic stresses in tomato
Source: Front Microbiol. 2023 Jun 9;14:1180538. doi: 10.3389/fmicb.2023.1180538 (PMC10288847; doi:10.3389/fmicb.2023.1180538)
Supplement: Supplementary file 1 [file Data_Sheet_1.PDF]

Supplemental Information

for

Endophytic *Streptomyces* Population induced by L-Glutamic Acid Enhances Plant Resilience to Abiotic Stresses

Da-Ran Kim[1], Youn-Sig Kwak[1,2]*

This file includes 4 figures.

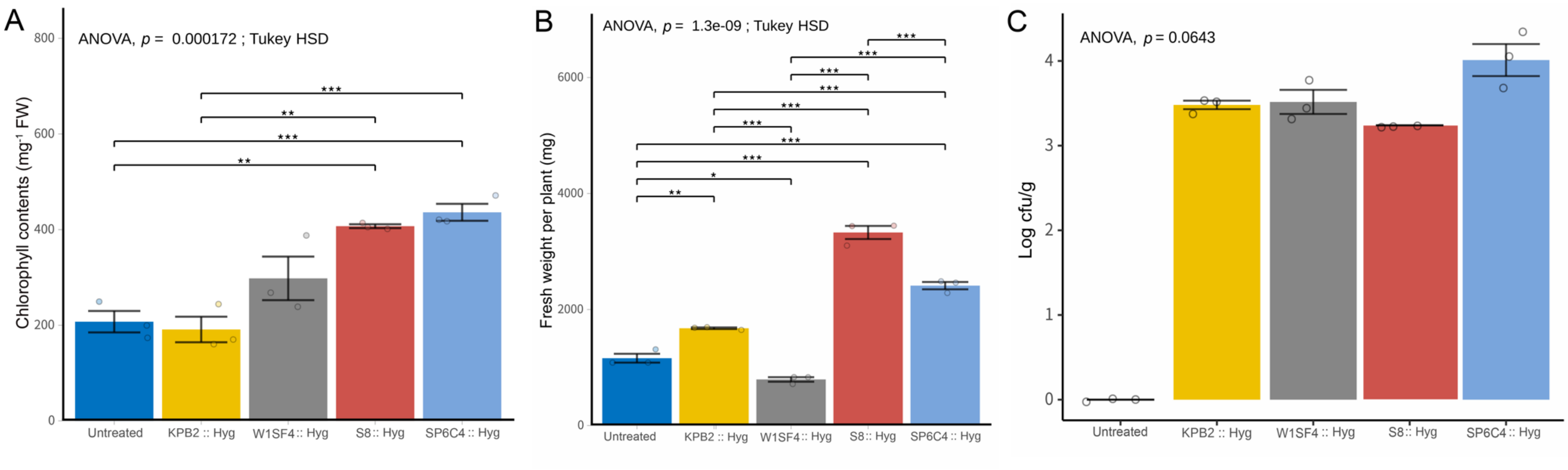

Fig. S1. Growth enhancement of tomato by *Streptomyces* strains and the bacterial population densities in the rhizosphere. *Streptomyces* spp. were cultured in PDK broth media supplemented with spring water for 10 days at 28°C in a shaking incubator. The cultured bacteria were mixed with 1% CMC, resulting in a final concentration of 0.1%, and coated onto tomato seeds, which were grown at an early vegetative stage for 2 weeks. The plant phenotypes and bacterial colonization in the rhizosphere were then measured. **A,** Chlorophyll contents were determined using a spectrophotometer with 1 mg of fresh leaves (n = 5). **B,** Fresh shoot weight was calculated for each plant (n = 5). **C** The bacterial colonization at the early vegetative stage was assessed by sampling the root and collecting root surface bacterial cells with MES buffer per 1 g of root (n = 5). All figures were tested using the Shapiro-

Wilk test ($p > 0.05$) and ANOVA test.

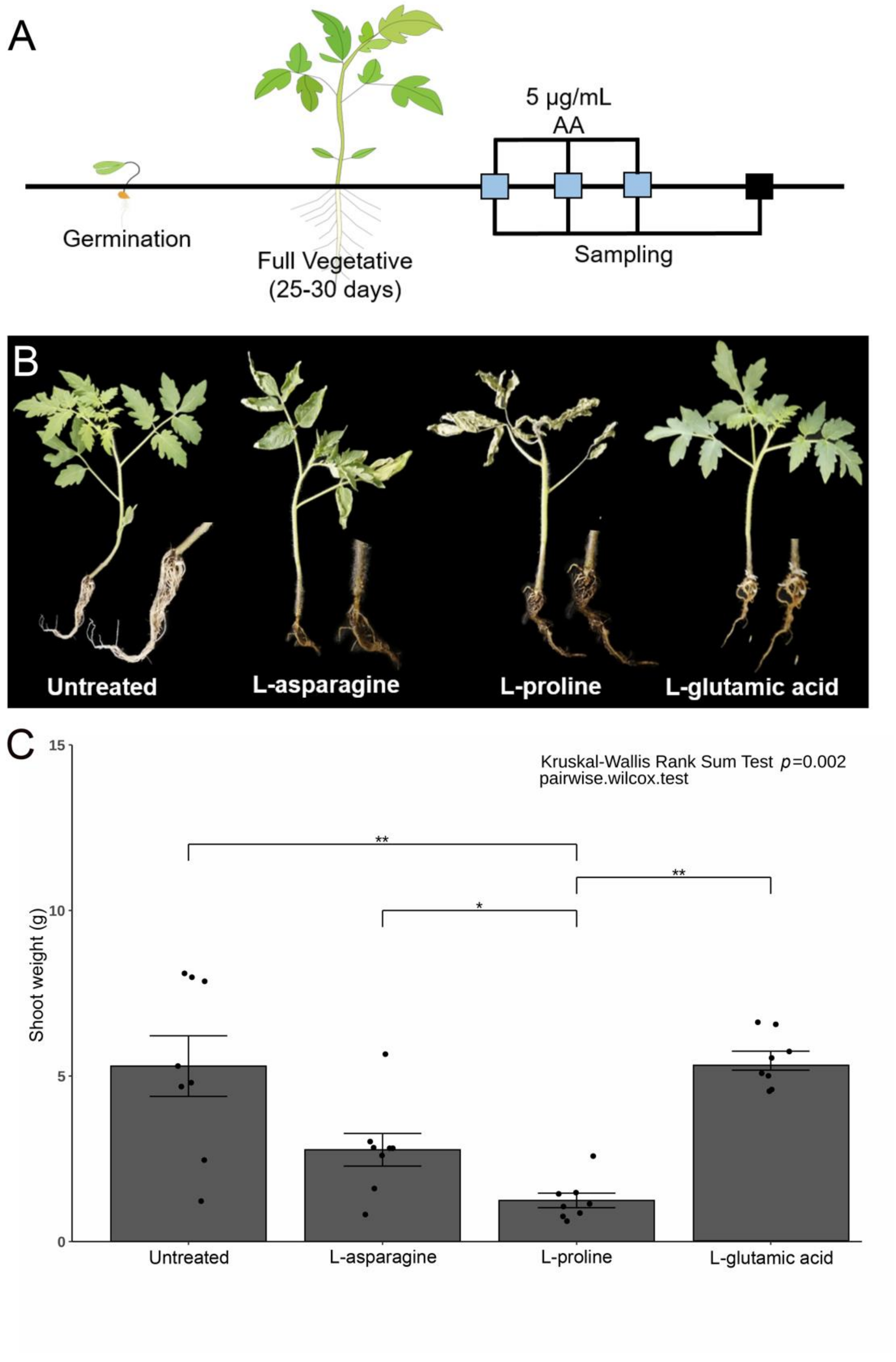

Fig. S2. Effect of amino acids on tomato growth. **A,** Schematic diagram of the experimental design used to study the effect of amino acid treatment on tomato plants. Three types of amino acids (L-asparagine, L-proline, and L-glutamic acid) were

applied at a concentration of 5 μg/mL during the vegetative stage. The amino acid stock was drenched into the tomato soil and applied once a week for 3 to 6 weeks. Untreated plants were watered with 1X Hoagland solution. **B,** After 6 weeks, the shoot length was measured, and the plant phenotype was observed by sampling 10 plants. **C** Statistical analysis was performed using the Shapiro-Wilk test, Leven's test ($p > 0.05$), and the Kruskal-Wallis test to compare the treatments.

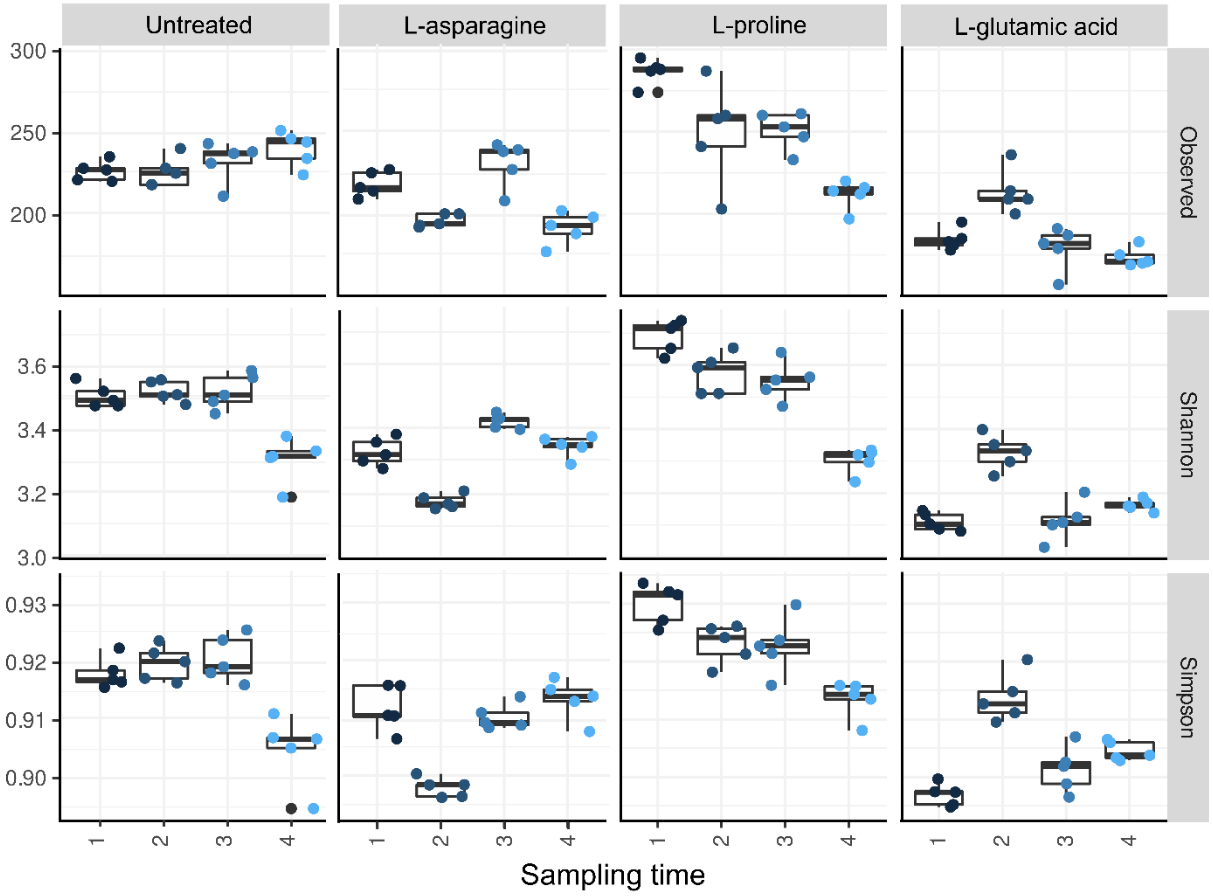

Fig. S3. Alpha diversity measurements of microbial diversity in the tomato root endosphere treated with amino acids. The species richness estimates were calculated based on the observed number of species, while the diversity estimates were determined using the Shannon and Simpson index. The boxes represent the first to third quartiles, the horizontal line in the box indicates the median and the upper and lower quartiles outside the box are also shown.

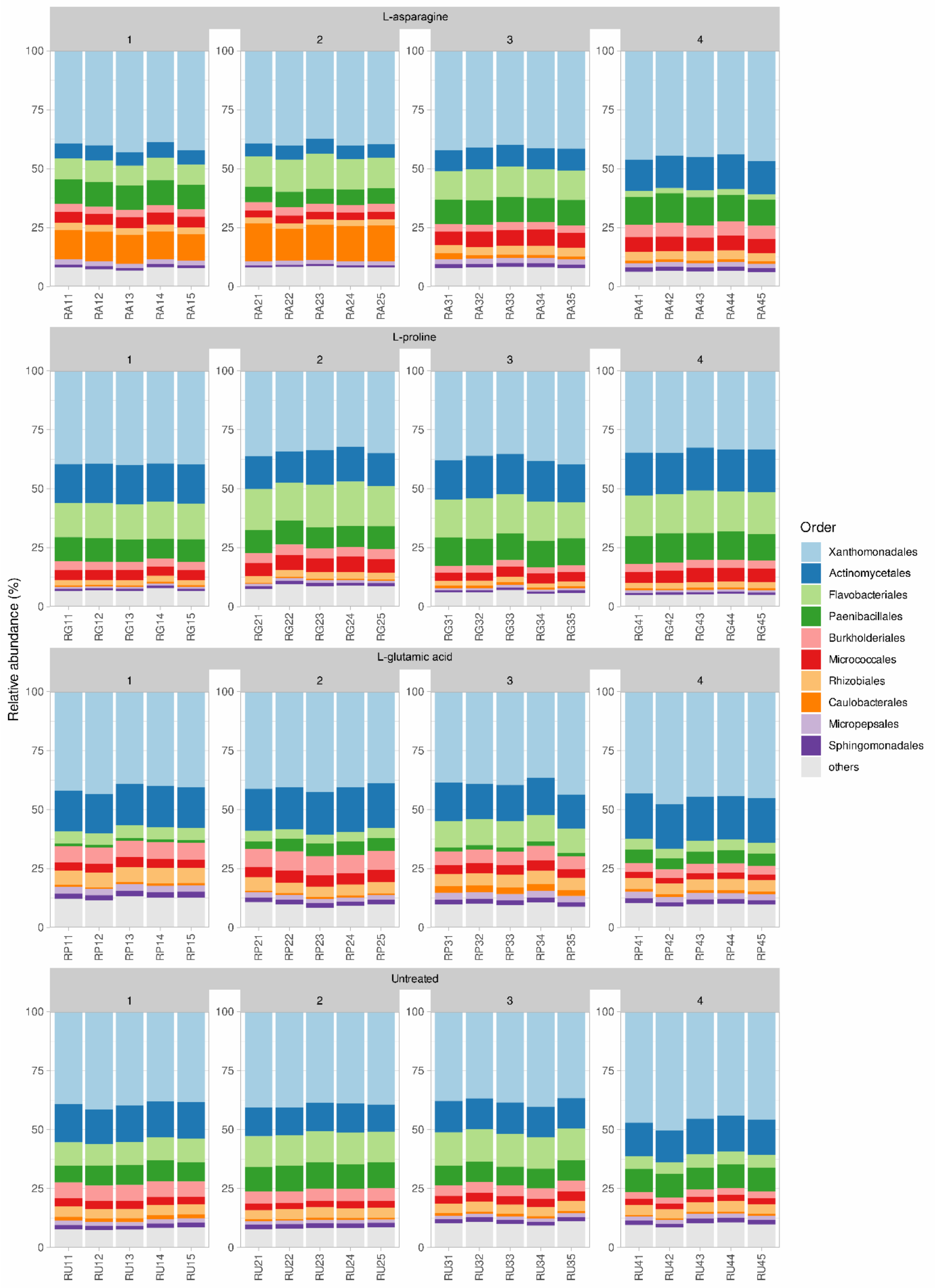

L-asparagine
L-proline
L-glutamic acid
Untreated
1
2
3
4
Relative abundance (%)
0
25
50
75
100
RA11 RA12 RA13 RA14 RA15 RA21 RA22 RA23 RA24 RA25 RA31 RA32 RA33 RA34 RA35 RA41 RA42 RA43 RA44 RA45
RG11 RG12 RG13 RG14 RG15 RG21 RG22 RG23 RG24 RG25 RG31 RG32 RG33 RG34 RG35 RG41 RG42 RG43 RG44 RG45
RP11 RP12 RP13 RP14 RP15 RP21 RP22 RP23 RP24 RP25 RP31 RP32 RP33 RP34 RP35 RP41 RP42 RP43 RP44 RP45
RU11 RU12 RU13 RU14 RU15 RU21 RU22 RU23 RU24 RU25 RU31 RU32 RU33 RU34 RU35 RU41 RU42 RU43 RU44 RU45
Order
Xanthomonadales
Actinomycetales
Flavobacteriales
Paenibacillales
Burkholderiales
Micrococcales
Rhizobiales
Caulobacterales
Micropepsales
Sphingomonadales
others

48

Fig. S4. Stacked bar plots depicting the relative abundance of the bacterial community in different amino acid treatments. The microbial beta diversity composition of the top ten ASV groups at the order level was estimated for each replication sample (n = 5). The amino acid treatment is indicated at the top of the gray bar, and sampling times are indicated under the treatment groups. X-axis represents the sampling name, with RA indicating root endosphere with L-asparagine, RG indicating root endosphere with L-glutamic acid, RP indicating root endosphere with L-proline, and RU indicating untreated root endosphere. The samples were split into different amino acid groups and untreated, with the number indicating the sampling time during the cultivation of tomato (cv. Heinze) in the vegetative stage.
